# Testicular Cancer and Cryptorchidism

**DOI:** 10.3389/fendo.2013.00032

**Published:** 2013-03-20

**Authors:** Lydia Ferguson, Alexander I. Agoulnik

**Affiliations:** ^1^Department of Human and Molecular Genetics, Herbert Wertheim College of Medicine, Florida International UniversityMiami, FL, USA

**Keywords:** testis, cryptorchidism, testicular cancer, spermatogonial stem cells, somatic cell niche

## Abstract

The failure of testicular descent or cryptorchidism is the most common defect in newborn boys. The descent of the testes during development is controlled by insulin-like 3 peptide and steroid hormones produced in testicular Leydig cells, as well as by various genetic and developmental factors. While in some cases the association with genetic abnormalities and environmental causes has been shown, the etiology of cryptorchidism remains uncertain. Cryptorchidism is an established risk factor for infertility and testicular germ cell tumors (TGCT). Experimental animal models suggest a causative role for an abnormal testicular position on the disruption of spermatogenesis however the link between cryptorchidism and TGCT is less clear. The most common type of TGCT in cryptorchid testes is seminoma, believed to be derived from pluripotent prenatal germ cells. Recent studies have shown that seminoma cells and their precursor carcinoma *in situ* cells express a number of spermatogonial stem cell (SSC) markers suggesting that TGCTs might originate from adult stem cells. We review here the data on changes in the SSC somatic cell niche observed in cryptorchid testes of mouse models and in human patients. We propose that the misregulation of growth factors’ expression may alter the balance between SSC self-renewal and differentiation and shift stem cells toward neoplastic transformation.

## Introduction

Cryptorchidism, or maldescended testes, is a common clinical diagnosis in newborn boys and one of the strongest risk factors for infertility and testicular cancer (Hutson et al., 2010). The position of the cryptorchid testicle may vary and can be located in the abdominal cavity, inguinal canal, or subcutaneous cavity, which could determine the extent of the associated phenotype. While some populations are affected at a higher frequency than others, around 2–4% of boys are globally diagnosed with either unilateral or bilateral cryptorchidism (Barthold and Gonzalez, 2003).

About 10% of all cases of testicular germ cell tumors (TGCT) occur in men with a history of cryptorchidism (Mannuel et al., 2012). Thus, a disruption of a common regulatory pathway, for example, androgen signaling, might be an underlying reason for the association of cryptorchidism and TGCTs. An alternative explanation is that the abnormal testis position itself is directly responsible for infertility and germ cell tumorigenesis. Indeed, the causative role of an abnormal testis position in infertility has been demonstrated in several animal experimental models. The elevated temperature of the undescended testis inhibits the differentiation of spermatogonia resulting in an arrest of spermatogenesis, reduced seminiferous tubule size, germ cell depletion, and fibrosis. The link between cryptorchidism and TGCT is however less clear. The most common type of TGCT in cryptorchidism is seminoma. It is commonly accepted that the precursor cancer cells are pluripotent germ cells. Whether such cells are derived from primordial germ cells (PGCs) also known as gonocytes, that continue to proliferate or undergo improper differentiation (Skakkebaek et al., 1987) or are a result of spermatogonial stem cell (SSC) transformation may be debatable. The abnormal testis position dramatically alters the function of somatic cells providing the niche for SSC self-renewal and differentiation. We review here the epidemiologic, genomic, and experimental data that might explain the higher incidence and the causes of TGCT in cryptorchidism. Clinical aspects of this disease are beyond the scope of this review and can be found elsewhere (Isidori and Lenzi, 2008).

### Testicular descent

During embryonic development, the gonads differentiate from the genital ridges. After completion of sex determination, both ovary and testis remain in a high pararenal position attached to the body walls by a mesenterial ligamentous complex derived from the mesonephric mesenchyme (Hutson et al., 1997; Barteczko and Jacob, 2000). At this stage, the cranial mesonephric ligament and the caudal genitoinguinal ligament (or gubernaculum) connect the gonads to the abdominal wall. The development and reorganization of these two ligaments, along with the differentiation of the epididymis, growth, and orientation of the gonads and reproductive tracts and finally the intra-abdominal pressure, direct the movement of the testis to the scrotum.

The two-stage model of testicular descent (Hutson et al., 1997), distinguishes the transabdominal phase characterized by the descent of the testis into the lower abdominal position, and the inguinoscrotal phase during which the testis moves through the inguinal canal and into the scrotum. During the transabdominal phase, the gubernacular cord and bulb are formed followed by the differentiation of muscle layers around the bulb. In humans, the first stage of testicular descent occurs between 10–15 weeks of gestation (Hutson et al., 1997). Transgenic studies in mice have identified INSL3 as the major factor in transabdominal testicular descent (Nef and Parada, 1999; Zimmermann et al., 1999; Overbeek et al., 2001; Gorlov et al., 2002; Huang et al., 2012) INSL3 is a small peptide hormone that belongs to the relaxin/insulin-like subfamily. It is expressed in testicular Leydig cells and is first detected right before the onset of testicular descent (Adham and Agoulnik, 2004). INSL3 signals through a G protein-coupled receptor called the Relaxin Family Receptor 2 (RXFP2). Transgenic overexpression of INSL3 in female mice leads to gubernaculum differentiation and ovary descent to a low abdominal position (Adham et al., 2002). Combined with the fact that an androgen deficiency does not affect transabdominal descent, one can assume that INSL3 is the primary peptide responsible for this process. Analysis of mutant gubernaculum development and the comparisons of gene expression in mutant and wild-type tissues performed in our laboratory, indicated that the NOTCH and WNT/beta-catenin cell signaling pathways might mediate the INSL3 effects at the cellular level (Kaftanovskaya et al., 2011). The effect of INSL3 deficiency is multifold; it causes suppression of myoblast differentiation in the muscle layers of the gubernaculum, apoptosis, and reduction of androgen receptor (AR)-positive cells within the base of the gubernaculum, and a failure of processus vaginalis development (Kaftanovskaya et al., 2011).

The second inguinoscrotal stage of testicular descent is clearly androgen-dependent (Hutson et al., 2010; Kaftanovskaya et al., 2012). Suppression of androgen production or AR deficiency has been linked to cryptorchidism in humans and various other species (Bay et al., 2011). Interestingly, an increase in testosterone production in human embryos precedes inguinoscrotal testicular descent (Hutson et al., 2010). The level of serum testosterone peaks at 15–18 weeks of fetal life and declines thereafter, whereas the testis remains in the same position for 5–10 weeks after the completion of transabdominal descent. In addition, several other processes occur during this time. Starting at about 8–10 weeks the scrotum anlage is formed and the floor of the gubernaculum base inverts to become the tunica of the sac-like processus vaginalis peritonei. The muscle layers at the rim of the gubernaculum further develop to become the wall of the cremasteric sac and the testes glide along the newly formed inguinal canal and into the scrotum (Van Der Schoot, 1996). Because the majority of these processes are androgen-sensitive, it is not surprising that many abnormalities that lead to a compromised differentiation or function of stereogenic Leydig cells as well as the failure of androgen signaling result in various degrees of cryptorchidism (Hutson et al., 1997).

A non-functional hypothalamo-pituitary-gonadal (HPG) axis results in hypogonadotrophic hypogonadism. The homozygous mutant mice for gonadotropin-releasing hormone (GNRH, *hpg*), GNRH-receptor (*Gnrhr*), and the LH receptor knockout mouse (*LuRKO*) devoid of LH stimulation, all have impaired inguinoscrotal testicular descent (Klonisch et al., 2004; Pask et al., 2005; Feng et al., 2006). A number of human syndromes related to testicular feminization or genitourinary dysplasia have the hallmarks of androgen deficiency as well (Klonisch et al., 2004). Estrogen-like and anti-androgen compounds have been shown to affect testicular descent through the suppression of the functional activity of Leydig cells and decreased testosterone and INSL3 production (Klonisch et al., 2004). Little is known, however, about local cell signaling pathways activated by androgen signaling in the developing gubernaculum, scrotum, cranial ligament, and epididymis. The link between inguinoscrotal cryptorchidism detected in the transgenic mouse and in some cases human mutants for several transcriptional factors, such as homeobox genes HOXA10 or HOXA11; Wilms tumor 1 (WT1); ARID domain-containing protein 5B (ARID5B), and androgen signaling in gubernaculum development still need to be determined (Klonisch et al., 2004; Kaftanovskaya et al., 2013).

### Epidemiology of cryptorchidism

The prevalence of cryptorchidism varies somewhat from country to country with the highest incidence reported in Denmark (9.0%) compared to that of the lowest, found in Finland (2.4%) (Boisen et al., 2004). Global cryptorchidism rates of around 2–4% are generally accepted (Barthold and Gonzalez, 2003). Several studies reported an increase in incidence during the 1970s and 1980s including Lithuania and the USA (Paulozzi, 1999; Preiksa et al., 2005), however in England rates have been declining since the 1990s (Jones et al., 1998).

Variations in rates of cryptorchidism over the years and also between countries such as the difference between Finland and Denmark, may reflect the impact of many contributing environmental factors. Man-made environmentally used chemicals such as pesticides, phthalates, bisphenol A (BPA), and polychlorinated biphenyls (PCBs) are known endocrine disrupters (Acerini and Hughes, 2006). Pregnant women treated with DES gave birth to boys with a higher incidence of cryptorchidism than non-treated women (Stillman, 1982).

Consumption of liver or smoked products, which have been found to contain a higher concentration of PCB has been implicated in higher rates of cryptorchidism (Giordano et al., 2008). Likewise, maternal consumption of more than five alcoholic drinks a week has been linked to an increase in the risk of cryptorchidism in one study (Damgaard et al., 2007), however disputed by others (Moller and Skakkebaek, 1997; Biggs et al., 2002; Kurahashi et al., 2005).

Similar disputes are also a factor in the contribution of maternal smoking to cryptorchidism. One study found that smoking more than 10 cigarettes a day during pregnancy (Jensen et al., 2007), increased the risk, whereas other studies have found no viable link (Mongraw-Chaffin et al., 2008). The discrepancies in the results of studies looking at maternal dietary and lifestyle factors may be a result of inconsistent data collection methods or inaccuracies from maternal questionnaires.

Given that the inguinal stage of testicular descent is between weeks 26 and 35, premature birth and low birth weight often associated with prematurity are contributing factors to an increased risk of cryptorchidism (Boisen et al., 2004; Jensen et al., 2007). However spontaneous correction is seen more frequently in boys who reach average weight within 1 year after birth, than in those who remain smaller (Preiksa et al., 2005; Jensen et al., 2007). Lifestyle factors that contribute to reproductive birth defects such as cryptorchidism may therefore have an indirect contribution to the formation of testicular germ cells in affected individuals.

### Germ cell tumors in cryptorchidism

Testicular cancer afflicts 1% of the male population and is the most common solid tumor to affect young men between the ages of 15–34. The association between cryptorchidism and TCGT has been well documented since the 1940s. Cryptorchidism is an accepted risk factor with a relative risk of 3.7–7.5 times higher than the scrotal testis population (Thorup et al., 2010). Conversely, it has been shown that 5–10% of men who develop testicular cancer, were or are cryptorchid (Thorup et al., 2010). There is an increased cancer risk in bilateral as opposed to unilateral cryptorchidism. Some studies have indicated that there is a direct correlation between how long the testis was subjected to a cryptorchid position and TGCT incidence. This can be seen from the data on surgical correction of cryptorchidism and the reduction of the risk of testicular cancer. One particular study found 13 out of 14 uncorrected cryptorchid patients between 1934 and 1975 developed TGCT in their abdominal testes (Batata et al., 1980). A Swedish group studied almost 17,000 men treated for cryptorchidism between 1964 and 1999 with the average age of surgery being 8.6 years. In this group, 56 individuals developed testicular cancer. Individuals who had corrective surgery before the age of 13 had an incidence rate of 2.23%, whereas those who were treated after 13 had an incidence rate of 5.4% (Pettersson et al., 2007). Based on such data in recent years, the recommended age of surgical correction was reduced and now is usually performed before the age of 2 (Pettersson et al., 2007). It should be mentioned however that some other reports did not find correlation between the time of surgery and risk of TGCT (Hack et al., 2007). In any case, even after early surgical correction the risk of TGCT is somewhat higher in patients with cryptorchidism.

The other factor that appears to play a role in TGCT incidence is the relative position of the cryptorchid testes, and hence the degree of environment insults on the gonads, such as heat. It was shown that an abdominal testis presents a greater risk for TGCT than an inguinal testis (Cortes et al., 2001).

Although corrective surgery has been found to reduce the risk from fivefold to twofold, in some cases the formerly cryptorchid testis becomes cancerous, indicative of permanent epigenetic changes in the cryptorchid testes (Hutson et al., 2010). Indeed, differences in promoter methylations and corresponding gene expression of several genes have been reported in TGCT. Apart from cell transformation such changes might be a result of environmental insults in cryptorchid testis. The other aspect that was extensively studied is the risk of TGCT in normally descended contralateral testes in men with unilateral cryptorchidism. Recent meta-analysis of such data indicated that the TGCT risk factor is much higher in affected testes than in scrotal one (6.33 vs. 1.74) (Akre et al., 2009).

It should be noted, that in many epidemiologic association studies the relative position of testes, age of surgical or spontaneous correction, presence of additional developmental abnormalities, or even variable definitions of cryptorchidism were not always taken into account. However a large majority of data indicates that age of surgery and the relative position of the cryptorchid testis are contributing factors to a greater risk of TGCT.

### Genetic factors in TGCT

The genetic factors and mutations involved in the potential predisposition to TGCT in the cryptorchid testis are not entirely clear as most studies do not differentiate between individuals with a history of cryptorchidism and those without. Genome-wide studies have identified six susceptibility loci, *KITLG* and *ATF7IP* on chromosome 12, *SPRY4* on chromosome 5, *BAK1* on chromosome 6, *TERT-CLPTM1l* on chromosome 5, and *DMRT1* on chromosome 9 (Rapley et al., 2009; Turnbull and Rahman, 2011). The KITLG-KIT pathway has been implicated in PGC development and is consistent with a role in contributing to TGCT (Kanetsky et al., 2009). SPRY4 a downstream target of the KITLG-KIT pathway and an inhibitor of the protein kinase pathway was linked to TGCT. Similarly, *BAK1* was also associated with TGCT in the same GWAS study. BAK1 is repressed by the KITLG pathway and acts as a germ cell apoptosis promoting factor by binding to the apoptosis repressor BCL2 (Turnbull and Rahman, 2011). Significantly, mice with mutations in *Kitl* genes require a specific genetic background to develop testicular cancer. Mice harboring a mutation of the *Steel* locus, which deletes *Kitl*, bred on a 129 background have a higher incidence of testicular cancer than wild-type controls. These mice also exhibit PGC defects in proliferation, migration, and survival demonstrating a possible link between pluripotent cell differentiation and TGCT (Heaney et al., 2008).

The telomerase encoding TERT locus and its transcription factor regulator ATF7IP are often overexpressed in cancers. Both alleles were associated with a predisposition for TGCT in a UK based whole genome association study (Turnbull et al., 2010). A similar study conducted in the USA, identified two SNPs within the DMRT1 allele – a zinc finger-like DNA-binding motif, significantly linked to TGCT. DMRT1 is expressed in the male gonad during Sertoli cell maturation and the deletion of this gene is associated with gonadoblastoma (Kanetsky et al., 2011).

Other genetic abnormalities were found to be associated with TGCT, however the causative nature of such mutations is not clear. Among these, the alleles of AR gene with short GGN repeats were linked to an increased risk of metastatic disease (Vastermark et al., 2011).

### Somatic mutations and gene misexpression

The role of somatic mutations in p53, PTEN, and other classical tumor suppressor genes in TGCT remains contradictory, however TGCT response to cisplatin-based chemotherapy indicates cancer cell sensitivity to p53 activation (Gutekunst et al., 2011). In some studies, a downstream target of p53, DAPK-1 was hypermethylated in seminomas compared to normal testes and was found to be clinically useful for testicular germ cell tumor stage diagnosis (Christoph et al., 2007). In contrast, a study that looked at 31 primary germ cell tumors found no mutations in p53 however 9 of 14 tumors tested positive with a p53 antibody (Lothe et al., 1995).

Platelet derived growth factors (PDGFs) and their receptors expressed in the pre- and postnatal testis, have also been implicated in testicular tumor formation (Basciani et al., 2010). An aberrant 1.5 kb transcript of the PDGF α-receptor was detected in TGCT and testis parenchyma with carcinoma *in situ* and was completely absent in normal testicular tissue. Moreover, this same aberrant transcript was detected in biopsies taken from cryptorchid testes containing CIS or germ cell tumor prior to corrective surgery. The expression of this transcript in TGCTs positively correlated with expression of the embryonic transcription factor OCT4/POU5F1. The chromosomal imbalances affecting the region containing *OCT3/4* and *KIT* genes involved in SSC maintenance, were also found in TGCT (Goddard et al., 2007; Gilbert et al., 2011).

Finally, another mouse model of TGCT is related to the overexpression of the growth factor glial cell line-derived neurotrophic factor (GDNF). GDNF is produced by Sertoli cells and targets GFRα1/RET co-receptors expressed by undifferentiated spermatogonia. It is well-established that this factor is crucial for self-renewal of SSCs. A mouse model expressing a full-length human GDNF transgene specifically in spermatogonia, began to develop tumors from 1 year of age (Meng et al., 2001). These tumors expressed the transgene, were derived from early germ cells, were alkaline phosphatase positive, and most closely resembled classical human seminomas.

Thus, several genes involved in SSC self-renewal, differentiation, and apoptosis were linked to TGCT. The association of these factors with cryptorchidism associated TGCT is less clear. It is important to note that none of the mouse mutants with an ablation of these genes exhibited cryptorchidism.

### Concept of testicular dysgenesis syndrome

First proposed back in 2001 (Skakkebaek et al., 2001), Testicular Dysgenesis Syndrome (TDS) suggests an existence of a developmental disorder resulting from a disruption of embryonic programing and gonadal development during fetal life. By definition it can be manifested as one or any combination of any of the four of the following developmental abnormalities: cryptorchidism, hypospadias, testicular cancer, and reduced semen quality. The current understanding of TDS includes both the hypothesis of a common environmental cause (Sharpe and Skakkebaek, 1993; Sharpe, 2003), as well as the existence of a common genetic factor responsible for all four abnormalities.

Some groups have reported an increase in the occurrence of cryptorchidism, hypospadias, infertility, and testicular cancer and several epidemiological studies indeed have shown an association of these symptoms. Skakkebaek et al suggested a role for an endocrine disruptor, responsible for the decline in male reproductive health (Boisen et al., 2001). During fetal development, such disruptors may have a detrimental effect on Leydig cells which could impair INSL3 or testosterone production, and thus affect gubernaculum development, testicular descent, or cause the malformation of the external male reproductive organs. Hormonal imbalance or direct endocrine disruptor effects on the Sertoli cells may also disrupt germ cell development resulting in infertility or TGCT. Thus, according to the TDS concept its symptoms are due to a common defect that affects a cell signaling pathway involved in a multitude of developmental and differentiation events in males. This might be the case in AR signaling deficiency, which has repercussions in testicular descent, infertility, and the masculinization of the genital tract (Miyagawa et al., 2009). Indeed, both experimental and epidemiologic studies show a link between cryptorchidism and infertility as well as an epidemiologic association between cryptorchidism and testicular cancer. It should be noted however that individuals presenting three or all four of the symptoms are extremely rare, and are usually associated with a 45X/46XY genotype or some forms of androgen insensitivity. Manifestations of one or two symptoms (such as cryptorchidism and infertility, or cryptorchidism and testicular cancer) are much more common.

In recent years, the common entity hypothesis of TDS has been the subject of debate. The critical evaluation of epidemiologic studies has brought into question the existence of widespread TDS due to the absence of non-casual associations between its different manifestations (Akre and Richiardi, 2009; Thorup et al., 2010). Most affected individuals exhibit one or two features, bringing into question whether one factor may be accountable for the occurrence of the abnormalities with such vastly diverse developmental etiology. On the other hand, the experimental data indicate that causative relationships can explain some of the epidemiologic correlation.

Since the TDS concept was proposed multiple attempts have been undertaken to identify genetic factors responsible for such a syndrome. The most recent genome-wide study investigated genetic variants affecting Danish males with at least one symptom of TDS in an attempt to attribute symptoms to a specific region of the genome. The study identified an association of subsets of these symptoms to genetic factors, in particular between cryptorchidism and testicular cancer (Dalgaard et al., 2012). The analysis highlighted a single nucleotide polymorphism in TGFBR3 mildly associated with all four symptoms of TDS. This gene encodes the TGFβ receptor type III found to be expressed in testicular Leydig cells and peritubular cells and when silenced in mouse, impedes Leydig cell function and normal cord formation (Sarraj et al., 2010). A member of the TGFβ superfamily, BMP7 was found to contain genomic variants in some patients with TDS symptoms, most notably those with cryptorchidism and testicular cancer. Likewise, mutations in the KITLG locus were mostly closely linked to an increased risk of testicular cancer and were not connected to any other TDS symptoms. Mutations in this gene have previously been associated with infertility as well as germ cell tumors (Galan et al., 2006). It should be pointed out however, that currently no mouse mutations in the genes encoding members of BMP/TGFβ signaling are known to cause isolated cryptorchidism.

The identification of a single or pairs of features of symptoms of TDS, as opposed to finding all four symptoms consistently running together, has further fueled speculation that the phenotypes of TDS are not due to a single cause. The occurrence of testicular cancer has increased over the last 40 years in Western countries along with rates of infertility, however rates of cryptorchidism remain unchanged. Hypospadias, most commonly associated with low birth weight, have shown a marginal increase in some studies since the 1950s but show wide epidemiological variation between countries. Interestingly, hypospadias are found isolated from cryptorchidism in 95% of cases and men with hypospadias alone do not suffer from reduced semen quality unless their symptom is accompanied with cryptorchidism. A much greater correlation between an undescended testis and a high risk of infertility or testicular cancer is supported by the alleviation offered by early orchiopexy. With 5% of germ cell tumors arising in previously cryptorchid testes, it is apparent that many genetic and pathologic factors interplay in each feature associated with TDS (Thorup et al., 2010).

### Cryptorchidism as the underlying cause of infertility and germ cell apoptosis

An alternative explanation to TDS, at least, with regard to cryptorchidism, is that the undescended testis itself causes an increased disposition for infertility and spermatogonial arrest, which in some cases may lead to abnormal germ cell differentiation and the formation of TGCT. This notion is strongly supported by the beneficial effects of surgical correction of cryptorchidism on future infertility and cancer risk. Experimental animal data also support this possibility. Induced cryptorchidism in animal models has been shown to lead to a depletion of germ cells, eventually resulting in infertility (Agoulnik et al., 2012), whereas orchiopexy restored spermatogenesis in several cryptorchid mouse mutants (Bogatcheva and Agoulnik, 2005).

The cause of germ cell depletion is strongly linked to the elevated environmental temperature of 37°C within the body cavity compared to the optimal temperature of 32°C for germ cells in the scrotum, although the mechanism behind this cause is not clear (Kumagai et al., 2000; Yin et al., 2002; Izu et al., 2004). Later stage haploid germ cells have been shown to be the most susceptible germ cells when exposed to abdominal temperature.

Apoptosis in the testis is essential for the establishment and maintenance of germ cell populations. In the cryptorchid testis, temperature induced apoptosis is responsible for the depletion of germ cells, however the molecular mechanisms behind this have not been fully determined. In the p53 knockout model, apoptosis is delayed by 3 days, from day 7 to 10 in the cryptorchid induced mouse, compared with the wild-type cryptorchid group. Thus it can be deduced that p53 is responsible for the initial phase of apoptosis in the germ cells (Yin et al., 1998). However, the initiation of apoptosis from day 10 is indicative of a p53-independent pathway after the initial phase of germ cell loss. In p53^−/−^ and lpr/lpr double mutant mice, apoptosis is further delayed compared to the cryptorchid control group, showing that Fas is responsible for the later stage germ cell loss (Yin et al., 2002). In addition, the occurrence of testicular apoptosis despite the delay, suggests that a third pathway is activated in the cryptorchid testis.

### Somatic cell changes in cryptorchidism

Spermatogonial cells are less affected by higher temperatures than all other germ cells and in the cryptorchid testis they survive the longest, however with time they are also eventually depleted. The question then arises, why do the spermatogonia cells decrease in the cryptorchid testis? Can it be linked to the changes in the somatic cell niche in seminiferous tubules?

The abnormal effects of the intra-abdominal environment in cryptorchid testes are not limited only to germ cells; somatic cells have also been shown to display dramatic changes in morphology, function, and gene expression. Sertoli cell vacuolization and abnormal cell adhesion are some of the most common consequences of cryptorchidism. Sertoli cells in an induced cryptorchid testis of the Rhesus monkey were found to have significant changes in the cytoskeleton (Zhang et al., 2004). Reactivation of cytokeratin 18 in adult Sertoli cells and increased expression of vimentin with disorganized staining characterized the heat stressed Sertoli cells in these primates with similar findings in the Sertoli cells of experimentally induced cryptorchid testes in rats (Wang et al., 2002).

In another study, rats with induced cryptorchidism showed disrupted actin filaments in the basal junction regions of the Sertoli cells compared to the regular lattice structures seen in the control rats (Maekawa et al., 1995). The morphological changes in the somatic cells of cryptorchid testes were also accompanied by changes in multiple genes’ expression of these cells.

In mice, Ribosomal Binding Protein Motif 3 (Rbm3) expression in adult Sertoli cells was reduced within 12 h of induced cryptorchidism (Danno et al., 2000). Likewise, the expression of FSH and ARs was dramatically reduced in cryptorchid lambs (Monet-Kuntz et al., 1987). While surgical correction restored their expression, up to 50% of seminiferous tubules did not recover, indicative of cryptorchidism-induced permanent damage.

Hadziselimovic et al. (2011) looked at differentially expressed transcripts in testis RNA isolated from patients with cryptorchidism with or without type A_d_ spermatogonia (A dark spermatogonia are believed to be SSCs in human) and therefore at a low or a high risk of developing azoospermia later in life. In the latter group, a number of genes involved in A_d_ spermatogonia self-renewal were not expressed, but were expressed in patients with a low risk and in the control groups. These factors included FGF9 which is essential for SSC self-renewal and acts as an inhibitor of meiosis through regulation of pluripotent genes, and FGFR3, expressed in prepubertal spermatogonia in the control and completely absent in the high risk group (Hadziselimovic et al., 2011).

Disruption of the blood-testis-barrier (BTB) enforced by Sertoli cells has also been reported in cryptorchid mice by breakdown of inter-Sertoli cell tight junctions which may have repercussions on the later stage germ cells. It was recently shown that cystic fibrosis transmembrane conductance regulator (CFTR) which enhances Sertoli cell tight junctions is significantly downregulated in cryptorchid testes and may contribute to the disruption of the BTB (Chen et al., 2012). This was also supported by the discovery that incubation of primary Sertoli cell cultures at 37°C, results in a decrease of CFTR expression compared to those incubated at 32°C. Breakdown of the BTB in both cases was shown by diffusion of injected tracker dye into the interstitial space (Chen et al., 2012). The physiological separation between pre and post meiotic germ cells, maintained by Sertoli cells in the testis, ensures that regulatory products are targeted to specific germ cell populations. Disruption of their cellular morphology, BTB, and expression of growth factors may lead to the misregulation of germ cells and abnormal differentiation, potentially leading to the formation of TGCTs.

### Origin of TGCT

The TGCT most commonly associated with cryptorchidism is seminoma. It is generally accepted that the classical seminomas develop from a precursor lesion, intratubular germ cell neoplasia (or carcinoma *in situ*, CIS). It is proposed to develop *in utero* from PGCs or early gonocytes (Skakkebaek et al., 1987). After remaining quiescent during infancy, the CIS is thought to proliferate at puberty and later progress to an invasive disease under the influence of factors such as gonadotrophins and/or testicular steroids (Skakkebaek et al., 1987). Contrary to the classical seminoma, the spermatocytic seminoma is believed to derive from differentiating spermatogonia. Recent data however showed that CIS and spermatocytic seminoma share a number of common markers with embryonic stem cells, for example, KIT, OCT3/4, SOX17, LIN28, NANOG, FGFR3, DMTR1, and others (Looijenga et al., 2003; Rajpert-De Meyts et al., 2003; Almstrup et al., 2004; Houldsworth et al., 2006; Gillis et al., 2011; Ryser et al., 2011). Thus, according to this hypothesis, expression of stem-related markers is a reflection of their origin from gonocytes. It should be pointed out however, that the expression of some of these genes is maintained and characteristic for adult SSCs (Waheeb and Hofmann, 2011).

The alternative theory of CIS transformation initiated during meiosis postulates that the common duplication of chromosome 12p in TGCT occurs due to abnormal recombination during meiosis (Chaganti and Houldsworth, 1998). The amplification of genes in this genomic region including *NANOG*, *DPPA3*, *GDF3* may provide a selective proliferation advantage and subsequent reactivation of a stem-like phenotype (Houldsworth et al., 2006).

It is difficult to test experimentally which model is correct. One indirect approach is to evaluate the incidence of CIS as a precursor TGCT in cryptorchid boys. If CIS is derived from embryonic gonocytes then the CIS should be detectable long before cancer development. In a study by Cortes et al. (2004), one invasive TGCT and six CISs were found in testicular biopsies of 182 cryptorchid patients with intra-abdominal testes, abnormal genitalia, and/or abnormal karyotype, but no cases were found in any of the 1281 cryptorchid patients without these additional characteristics. The absence of neoplasm detection in patients with isolated cryptorchidism might therefore indicate that CIS and TGCT are derived from adult germ cells.

The SSC niche is tightly regulated between proliferation and differentiation by factors supplied by testicular somatic cells. GDNF acts upon the spermatogonial receptors RET and GFRα1. GDNF is critical for self-renewal; overexpression of GDNF in mouse spermatogonial cells leads to the formation of germ cell tumors (Meng et al., 2001), whereas a GDNF knockout model results in an absence of germ cells (Naughton et al., 2006). Activation of GDNF and RET/GFRα1 complex leads to the activation of two signaling pathways, which in turn up-regulate the transcription factors MYCN (Braydich-Stolle et al., 2007) and FOS (He et al., 2008). GDNF has also been linked to the upregulation of FGF2 and it was demonstrated that the addition of FGF2, along with GDNF and GFRα1 to a serum-free culture of SSCs, provided the optimal conditions for SSC colony number (Kubota et al., 2004). Long-term survival and proliferation of SSCs in culture have been tested with different concentrations and complements of growth factors. High concentrations of leukemia inhibitory factor (LIF) and FGF2 were found to have a detrimental effect on the colonization of SSCs (Kubota et al., 2004). KITLG secreted by Sertoli cells and also found to be mutated in some seminomas, facilitates the differentiation of spermatogonial cells (Pellegrini et al., 2008). In addition, colony-stimulating factor (CSF) secreted by Leydig cells and its receptor CSF1R located on undifferentiated spermatogonia have also been implicated for the sufficient proliferation of spermatogonia (Kokkinaki et al., 2009).

**Figure 1:**
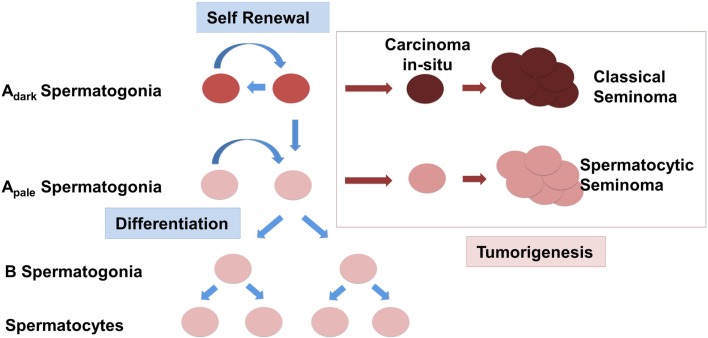
**Schematic representation of the origin of testicular germ cell tumors (TGCTs) in humans**. Normal spermatogonial stem cells (SSCs) A_dark_ and A_pale_ either undergo a cycle of self-renewal or begin to differentiate and are controlled by the expression of somatic growth factors. Classical seminomas are derived from carcinoma *in situ* cells (CIS). Spermatocytic seminomas originate from differentiating spermatogonia.

Changes in the expression of somatic factors in cryptorchid testes in humans have been detected using whole genome studies as previously mentioned, and interestingly, many of these differentially expressed genes such as NY-ESO, FGFR3, UTF1, and DSG2 are aberrantly expressed in seminomas (Waheeb and Hofmann, 2011). One study found that ERK1/2 – an intermediate of the GDNF pathway that leads to activation of FOS and other target genes, was increasingly phosphorylated in more than half of 26 seminomas potentially altering expression of downstream targets of the RAS/ERK1/2 pathway including FOS and ATF (Goriely et al., 2009). Taken in conjunction with the finding that GDNF overexpression in mice leads to the formation of seminomas in advanced age, it is conceivable to suggest that changes in somatic cells in the testis can lead to the deregulation of the SSC somatic niche (Clark, 2007; Kristensen et al., 2008). Instead of self-renewal and differentiation, SSCs in this scenario will be preferentially forced into the differentiation pathway (Figure 1). In an abnormal cryptorchidism-induced somatic niche environment most of the differentiating germ cells will be eliminated due to arrest of spermatogenic differentiation, lack of proper cell signaling, abnormal cell junction, and increased apoptosis. The depletion of the SSC pool will eventually lead to the complete loss of germ cells in the cryptorchid testis, which would explain the infertility in patients with surgical correction performed later in life. Accumulation of additional mutations or chromosome rearrangements, including amplification of chromosome 12p, might provide a selective growth advantage for SSC or differentiating germ cells. Instead of the spermatogenetic pathway the cells can now escape into a pre-neoplastic state. Depending on the differentiation stage when such an event occurs, this might give rise either to CIS further progressing to a classical seminoma or the development of a spermatocytic seminoma, derived from more differentiated spermatogonial cells. This scenario does not rely on a common factor causing two seminoma phenotypes with dramatically different etiology, but rather defines cryptorchidism only as an independent causative risk factor in the development of TGCT.

## Conclusion

Testicular cancer is accountable for 1% of all cancers in men and is the most common in men between the ages of 15 and 34. The risk of developing testicular cancer due to a cryptorchid testis is increased to 5–10 times that of the general male population. This increased risk for a cryptorchid or previously cryptorchid individual is indicative of long-term damage, despite early orchiopexy in many cases. Very little is known about mechanisms of TGCT tumorigenesis and at present there is no animal model that develops testicular cancer as a result of an undescended testis phenotype. Gene expression studies on cryptorchid patients and animal models have indicated that growth factors known to be important for the balance of self-renewal and the proliferation of germ cells are deregulated. The aberrant testicular environment also has a detrimental effect on Sertoli and Leydig cells that may lead to an inability to support the stem cell population. Accumulation of mutations in the somatic cells may lead to misexpression of important growth factors and morphological breakdown. Consequently, in the cryptorchid testis, an alternative differentiation pathway for SSCs is proposed which can result in the formation of TGCT. Identification of new markers specific for fetal germ cells and SSCs in human patients will help to delineate the origin of TGCT. The detailed analysis of changes induced in the SSC somatic cell niche by cryptorchidism and the fate of SSCs will be crucial for our understanding of the link between cryptorchidism and TGCT.

## Conflict of Interest Statement

The authors declare that the research was conducted in the absence of any commercial or financial relationships that could be construed as a potential conflict of interest.
